# Improve the Strength of PLA/HA Composite Through the Use of Surface Initiated Polymerization and Phosphonic Acid Coupling Agent

**DOI:** 10.6028/jres.116.021

**Published:** 2011-10-01

**Authors:** Tongxin Wang, Laurence C. Chow, Stanislav A. Frukhtbeyn, Andy Hai Ting, Quanxiao Dong, Mingshu Yang, James W. Mitchell

**Affiliations:** College of Engineering, and College of Dentistry, Howard University, Washington, DC 20059; American Dental Association Foundation, Paffenbarger Research Center, Polymer Division, National Institute of Standards and Technology, Gaithersburg, MD 20899; College of Engineering, Howard University, Washington, DC 20059; College of Engineering, and College of Dentistry, Howard University, Washington, DC 20059; Institute of Chemistry, Chinese Academy of Sciences, Beijing 100190, China; Institute of Chemistry, Chinese Academy of Sciences, Beijing 100190, China; College of Engineering, Howard University, Washington, DC 20059

**Keywords:** bioresorbable, composite, hydroxyapatite, interface, mechanical, PLA, polymerization

## Abstract

Bioresorbable composite made from degradable polymers, e.g., polylactide (PLA), and bioactive calcium phosphates, e.g., hydroxyapatite (HA), are clinically desirable for bone fixation, repair and tissue engineering because they do not need to be removed by surgery after the bone heals. However, preparation of PLA/HA composite from non-modified HA usually results in mechanical strength reductions due to a weak interface between PLA and HA. In this study, a calcium-phosphate/phosphonate hybrid shell was developed to introduce a greater amount of reactive hydroxyl groups onto the HA particles. Then, PLA was successfully grafted on HA by surface-initiated polymerization through the non-ionic surface hydroxyl groups. Thermogravimetric analysis indiated that the amount of grafted PLA on HA can be up to 7 %, which is about 50 % greater than that from the literature. PLA grafted HA shows significantly different pH dependent ζ-potential and particle size profiles from those of uncoated HA. By combining the phosphonic acid coupling agent and surface initiated polymerization, PLA could directly link to HA through covalent bond so that the interfacial interaction in the PLA/HA composite can be significantly improved. The diametral tensile strength of PLA/HA composite prepared from PLA-grafted HA was found to be over twice that of the composite prepared from the non-modified HA. Moreover, the tensile strength of the improved composite was 23 % higher than that of PLA alone. By varying additional variables, this approach has the potential to produce bioresorbable composites with improved mechanical properties that are in the range of natural bones, and can have wide applications for bone fixation and repair in load-bearing areas.

## 1. Introduction

Bioresorbable materials, including polymers and their composites, are desirable for bone fixation and repair applications because the materials do not need to be removed by surgery after the bone heals as is the case with metallic implants. Representative bioresorbable materials that are approved for clinical use include polylactide (PLA) and its copolymer poly(lactic-co-glycolic acid) (PLGA), but polymers alone usually have the adverse clinical effect that the degraded acidic monomers may cause inflammatory or allergic reactions [1]. Incorporating osteoconductive fillers such as hydroxyapatite (HA) into the PLA matrix is a useful way to improve clinical performance. Because of the similarity in composition to that of bone and teeth, introduction of HA into PLA not only neutralizes the degraded acidic products of PLA, but also makes the implant more osteoconductive, thus improving tissue compatibility [2–3].

To date, a large number of reports have been published on the preparation of PLA/HA composites. However, a critical problem of current PLA/HA composites is their weak mechanical properties, which do not allow them to be used in load-bearing areas. Generally, the mechanical properties of composites are determined by a number of factors including the inherent characteristics of the filler (crystallinity, size, shape) and the matrix (relative molecular mass, chemical structure, configuration), volume fraction, dispersion of the fillers within the matrix, integrity of the interface, and the interfacial bond strength [4]. For a given combination of PLA and HA materials, optimizing the filler-matrix interface should be a key factor to achieving improved mechanical strengths, especially when the filler has a large surface area, such as in the case of micro- or nano-fillers.

Current PLA/HA composites are prepared by several different methods such as direct blending using non-modified HA, solution co-precipitation [5–7], emulsion [8], and mechanical mixing [9–10]. Because of the relatively high hydrophobicity of PLA and hydrophilicity of HA [11–12], obvious problems of these methods include weak interfacial adhesion between HA and the PLA matrix and agglomeration of the HA particles in the matrix. Lack of adhesion between the two phases will result in an early failure at the interface, usually leading to weak mechanical properties [12–14]. As an example, the tensile strength of PLA/HA composites decreased significantly from 54 MPa for pure PLA to 41 MPa even with a HA content of only 18 % [15].

In the last decade, coupling agents such as silanes [16], isocyanates [17], and organotitanates [18] have been used to improve the interfacial adhesion between the ceramic filler and polymeric matrix, Although the effects on alumina and silica systems (SiO_2_, bioglass, clay, etc.) were significant [19–20], the feasibility of using these agents to gain improved interfacial adhesion to HA was not confirmed [21]. In particular, because the coupling reaction of silane or isocyanate with HA is based on the surface –OH groups, these agents may not be suitable for HA due to the limited number and reactivity of surface –OH groups on HA.

Recent research on biomimetic mineralization [22] and dental materials [23] suggests that phosphonic groups can have a strong adhesion to calcium phosphates. A number of phosphorous compounds have been used in calcium phosphate-based biomaterials such as HA-phosphonate composites [24], phosphorylated chitosan coated calcium phosphate [25], and dental bonding agents (Ivoclar Vivadent^1^) [23]. It would therefore be logical to explore the possibility of using phosphorus compound as a coupling agent to enhance interfacial adhesion between HA and PLA.

Grafting polymer onto the filler is also an effective way to improve the interfacial adhesion because it can establish direct chemical bonding between the polymer and the filler [26]. Polymers may be grafted to the filler surface either through an end-functionalized polymer (grafting to) or a surface initiated polymerization (SIP) (grafting-from) approach [27]. An advantage of ‘grafting-from’ methodology is that all of the polymers will be covalently linked with the particle. SIP has been used to prepare calcium phosphate composites with non-resorbable polymers such as PMMA (methyl methacrylate)[28] and PVPA (vinyl phosphonic acid) [29]. At present, bioresorbable polymers such as polycaprolactone (PCL)/HA [30] and PLA/HA have only been used with non-modified HA [15, 31] or lactic modified HA [19] for composite preparations. Because of the limited number of surface –OH on HA [15, 32] and its low reactivity, the use of the innate surface –OH on HA for SIP is unlikely to yield a sufficient amount of PLA graft. Thus, it would be desirable to explore an alternative approach that introduces surface –OH onto HA through a phosphonic coupling agent described above.

The approach evaluated in the present study consisted of two major steps. The first step was to produce a larger amount of –OH groups on the HA surface through a phosphonic based bi-dentate chelating agent that can bond firmly to the HA surfaces. This was followed by grafting PLA from the HA through the surface –OH groups provided by the phosphonic agent. The hypothesis to be tested was that the combination of chemically bonding the phosphonic group should increase the activity of surface –OH groups while SIP should improve the PLA/HA interface, leading to improved mechanical properties of the composite.

## 2. Materials and Methods

### 2.1 Materials

CaO, prepared by heating CaCO_3_ (reagent grade >99 %, Sigma-Aldrich, St. Louis, MO) at 800 °C overnight, was used for preparing the Ca(OH)_2_ slurry. L-lactide (98 %, Sigma-Aldrich) was recrystallized from dry ethyl acetate three times before used. Other reagents, including stannous octoate (SnOct_2_, 95 %, Aldrich), H_3_PO_4_ (reagent grade 85 %, Sigma-Aldrich), N-(2-hydroxyethyl) iminobis(methylphosphonic) acid (HIMPA) solution (≈ 50 % in H_2_O, Sigma-Aldrich), and polylactide (PLA) (average relative molecular mass 99 000, Fluka) were used as received.

### 2.2 Preparation of HA Using a Solution Precipitation Method

HA was synthesized by solution reaction of Ca(OH)_2_ and H_3_PO_4_ in accordance with the method of McDowell as described in Markovic et al. [33]. In brief, about 500 mL of distilled water was boiled in a Teflon-coated pot, equipped with an electric stirring paddle and a reflux condenser with a CO_2_-absorbing NaOH trap to protect from atmospheric CO_2_, under Ar gas for 60 min. One mole of CaO was added to the water, and 300 mL of H_3_PO_4_ solution (2 mol/L) was slowly (about 0.5 mL/min) added to the Ca(OH)_2_ slurry to obtain a final Ca/P molar ratio of 1.67. The reacting mixture was boiled for 2 d. The precipitated solid was collected by centrifugation and washed with distilled water. The solid was re-dispersed in boiled distilled water and was re-boiled for another 2 d. These washing and boiling procedures were repeated until the pH of the supernatant was ≈ 6. At pH 6, any traces of anhydrous dicalcium phosphate (DCPA) that might have formed due to possible local more acidic environments were converted to HA. In some cases, the HA precipitate, collected by centrifugation, was used for phosphonic acid coating without further processing as described below. In other cases, the HA precipitate was collected by centrifugation, washed with acetone, and dried at 110 °C.

### 2.3 Coating HA Particles Using Phosphonic Acid Based Agents

#### Method A. Coating HA particles using surface modification [34]

The HA particles, freshly precipitated without drying, were suspended in an aqueous solution of HIMPA (2.5 %) at a HA/HIMPA mass ratio of 5:1. The coating suspension was adjusted to pH 7 and stirred overnight at room temperature under argon (Ar) to avoid absorption of CO_2_. Unreacted HIMPA was removed from the coated HA particles by repeated washing with distilled water followed by centrifugation. The particles were dried in a vacuum oven at 110 °C for 72 h.

#### Method B. Coating HA particles using in situ co-precipitation

Method B was an in situ co-precipitation method, in which HA was precipitated in the presence of HIMPA. This method was modified from that reported by Haque et al. [28] for HA synthesis and coating by co-precipitation. In this method, phosphonic acid groups were expected to partially substitute the phosphate groups on the HA surface. Thus, the reactant quantities were adjusted to account for this substitution, i.e., to obtain the Ca/(PO_4_^3−^ + PO_3_H_2_) molar ratio of 1.67 for HA, using a calculation similar to that described by Gibson et al. [35]. Briefly, calcium oxide (1.0 mol) was added to the 500 mL boiling water. One hundred mL of H_3_PO_4_ (5 mol/L) and 100 mL of HIMPA (0.5 mol/L) were added sequentially to the Ca(OH)_2_ slurry over 1 h. Because there are two phosphonic groups in one molecule of HIMPA, the effective molar ratio of Ca/(PO_4_^3−^ + PO_3_H_2_) was kept as 1.67. The reacting condition and purification procedures were the same as that for HA synthesis described above.

### 2.4 Grafting PLA on HA Particles Using Surface Initiated Polymerization (SIP) (Preparation of PLA-*g*-HA)

The grafting reaction of PLA on HA was performed according to the method of Hong et al. [15]. In an intensively flame-dried glass ampoule, 2 g of L-lactide was dissolved in 10 mL of dry toluene or dimethylformamide (DMF). The solvents, toluene and DMF, were dried freshly over sodium or calcium hydride, respectively. 2 g of HA that had been dried at 120 °C for 72 h in a vacuum-oven was suspended in 20 mL of dry toluene containing 10 μL of the SnOct_2_ catalyst in a flask. The suspension was heated to 90 °C and was dropped into the L-lactide solution under argon protection and with stirring. After the reaction continued at 140 °C for 48 h, the reaction mixture was cooled down to room temperature. The PLA-grafted-HA (PLA-*g*-HA) particles were separated by centrifugation and washed with excess volumes of methylene chloride five times to completely remove the free PLA that did not graft on the surface of the HA particles. Finally the separated sediment was dried in a vacuum-oven at 50 °C for 24 h to remove the residual solvent.

### 2.5 Preparation of PLA/HA Composite From PLA-*g*-HA and Additional PLA

PLA/HA composites were prepared according to the literature [15]. Briefly, 0.4 g of non-treated HA or PLA-*g*-HA and 1.6 g of PLA were dispersed in 40 mL of methylene chloride. The suspension was stirred vigorously for 3 h. The composite was precipitated from the suspension with an excess of ethanol (≈100 mL) and then dried in vacuum at 50 °C for 72 h to remove the residual solvent. The HA content of the composite was approximately 20 % mass fraction.

### 2.6 Characterization Methods and Mechanical Tests

#### Powder X-ray diffraction (XRD)

XRD (Rigaku DMAX 2200) was used to determine the crystalline phases and crystallinity of these phases present in the product [33]. Scans were performed between 10° < 2θ < 50°. The estimated standard uncertainty was 0.05°.

#### pH dependent particle size and surface charge (ζ-potential)

Median particle size (Z) and ζ-potential of pure HA and PLA-coated HA were measured by Zetasizer Nano ZS (Malvern). Samples were prepared by dispersing the HA powder in distilled water followed by sonication for 10 min. The starting suspension was prepared by adding 40 mg of HA in 12 mL of 0.1 mol/L NaOH (pH = 13). The measurements were conducted from pH 13 to pH 2 by adding 1 mol/L HCl as the titrant. Each sample was measured three times and the average value was taken. Unless stated otherwise, throughout the present study the standard deviation is considered as the estimated standard uncertainty of the measurement.

#### Thermogravimetric analysis (TGA)

TGA was conducted to understand the amount of HIMPA and PLA coated on the HA. The samples were dried at 110 °C for at least 72 h prior to TGA measurement. TGA was run on a TG/DTA320 instrument (SII Nano Technology, Inc.) from room temperature to 700 °C at a heating rate of 20 °C/min (in air). The amount of surface grafted PLA was determined as a weight loss percentage during heating with comparison to the weight losses of HIMPA coated HA and non-coated HA. The TGA measurements for each sample were conducted two or more times to establish reproducibility.

#### Diametral tensile strength measurements

The diametral tensile strength (DTS) was tested according to the previous reported method by Chow et al. [36]. The hot pressing mold used for preparing the specimens consists of a stainless steel cylinder with a 6 mm-diameter hole and two plungers of the same diameter. The specimens were prepared at 180 °C under 15 MPa for 60 min. A pre-determined mass of composite was used to obtain specimen with the dimensions of approximately 3 mm H × 6 mm D. The sample was placed between steel platens and tested on a Universal Testing Machine (United Calibration Corp, Garden Grove, CA) at a loading rate of 10 mm/min. All mechanical strength and modulus data were obtained by averaging over three specimens.

### 2.7 Statistical Analysis

One way ANOVA (analysis of variance) was used to analyze the data obtained from the mechanical tests.

## 3. Results

### 3.1 HA Preparation and Surface Coating With HIMPA

**Fig. 1:**
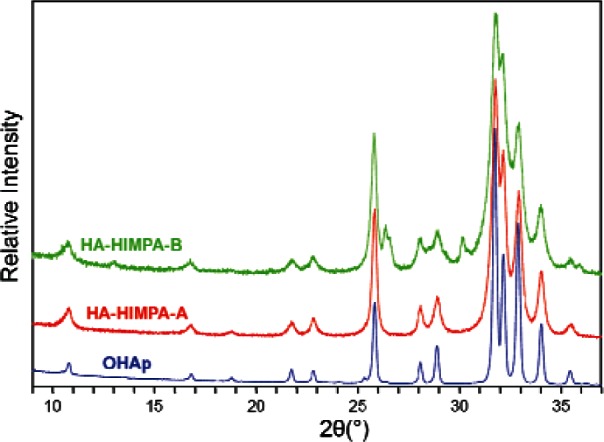
X-ray diffraction patterns of highly crystallized HA standard, and HA coated with HIMPA by two different methods: (1) HAHIMPA-A from method A—prepared by coating precipitated HA with HIMPA; and (2) HA-HIMPA-B from method B—prepared from co-precipitation of HA in the presence of HIMPA.

The XRD pattern of the as-prepared pure HA (Fig. 1) is typical for crystalline HA prepared by the precipitation method reported in the literature [33]. The pattern of the HIMPA coated HA by method A (HA-HIMPA-A) shows a very slight decrease in crystallinity compared to that of pure HA. In contrast, the HA-HIMPA-B pattern exhibits more discernable peak broadening, indicating significantly lower crystallinity, most probably a result of partial substitution of the phosphate in HA by the phosphonate.

**Fig. 2:**
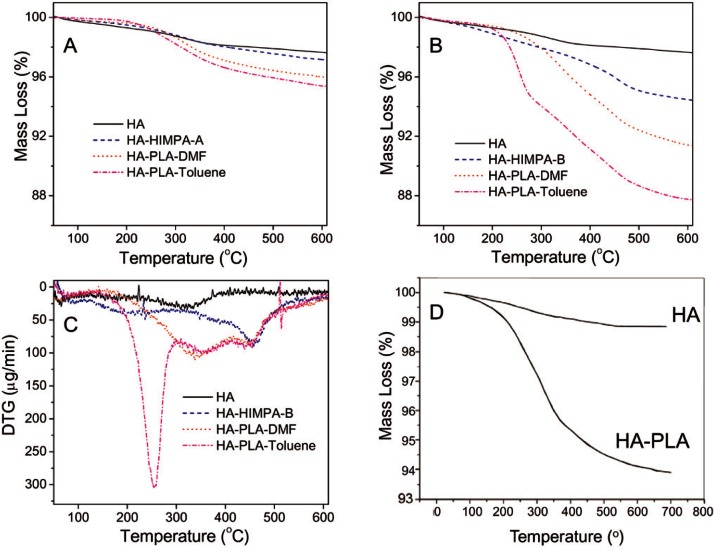
TGA curves of pure HA, HA coated with HIMPA (HA-HIMPA), PLA grafted HA (HA-PLA) in DMF or toluene solvent: (A) from method A, (B) from method B, (C) DTG curves of samples of B-series, and (D) TGA curves of HA and HA-PLA from reference [15].

TGA was conducted to estimate the amount of HIMPA coated on HA. Figure 2A shows the TGA curves of pure HA and HIMPA coated HA. Both the pure HA and HA-HIMPA from method A showed a similar 2.5 % mass loss when heated to 600 °C, implying that the amount of the HIMPA coating on HA using method A was too little to be observed by TGA. In comparison, HA-HIMPA-B showed a 5.5% mass loss (Fig. 2B), suggesting a significant amount of HIMPA coating by method B. Based on the mass loss differences among the pure HA, HA-HIMPA-A, and HA-HIMPA-B samples, the HIMPA content on HA-HIMPA-B was estimated to be about 3 %. These results suggest that phosphonic acid can be more effectively coated on HA using the in situ co-precipitation method B, probably by formation of a hybrid Ca/(PO_4_^3−^ + R − PO_3_^2−^) shell over the core of HA, as elucidated further below.

### 3.2 Surface Initiated Polymerization (SIP) of PLA on HA

The amount of PLA grafted on HA was also evaluated by TGA. Figure 2A shows the TGA curves for samples of PLA grafted onto HIMPA coated HA samples by method A (PLA-HA-HIMPA-A), using either DMF or toluene as the solvent. The mean values of mass loss for both PLA grafted samples were about 4 % mass fraction, which are greater than the 2.5 % mass loss for the pure HA and HA-HIMPA-A samples. This implies that a small amount of PLA (1.5 % mass fraction) can be grafted on HA either in DMF or toluene, with toluene being a somewhat better solvent.

Figure 2B shows the TGA curves for the sample series using the in situ co-precipitation method B. The mean values of mass loss at 600 °C of HA, HA-HIMPA-B, PLA-HA-HIMPA-B-DMF and PLA-HA-HIMPA-B-Toluene were 2.5 %, 5.5 %, 8.5 % and 12.5 %, respectively. Thus, the amounts of PLA coating on the HA particles prepared by method B in DMF and toluene were approximately 3 % and 7 %, respectively. These values are 2 and nearly 5 times, respectively, those produce by method A. It can be concluded that method B together with toluene as the solvent can efficiently produce a large amount of PLA onto the HA particles. This coating amount is significantly greater than that reported in the literature (5 %, Fig. 2D) [15], and is in support of our hypothesis stated earlier.

In order to gain further understanding of the characteristics of the samples, Fig. 2C shows the first derivatives of the TGA (DTG) curves of the same samples from Fig. 2B. The pure HA exhibited a relatively flat curve except for a broad peak around 320 °C. In contrast, HA-HIMPA-B shows a large peak around 450 °C, probably due to the loss of HIMPA that was incorporated within the HA. PLA grafted HA from both solvents showed the same mass loss profile around 450 °C. In addition, PLA HA-HIMPA-B-toluene shows a significant peak around 260 °C, which can be attributed to the loss of PLA. The sharpness of this peak provides evidence of a large amount of PLA on HA.

The above results indicate that phosphonic acid (HIMPA) can be used as an efficient coupling agent to coat HA particles, especially by using *in situ* co-precipitation of HA in the presence of HIMPA (method B). PLA can be grafted on HIMPA coated HA through surface initiated polymerization. Due to the greater amount and different kind of –OH groups on the HIMPA-HA than that of uncoated HA, more PLA can be grafted on HIMPA-coated HA by using method A or method B than on HA alone. Moreover, the amount of grafted PLA by method B is greater than that of method A, indicating that HIMPA coating produced by method B is a more efficient approach to graft more PLA onto HA particles.

### 3.3 Differences in Surface Characteristics of HA and PLA-Grafted-HA

**Fig. 3:**
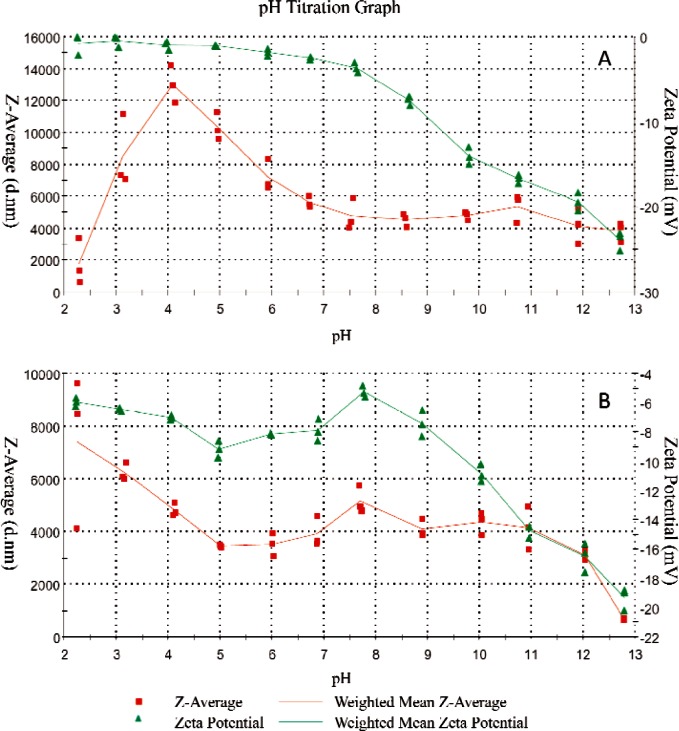
pH dependent mean particle size (Z, in nm) and ζ-potential (mV) of: (A) pure HA and (B) PLA-grafted HA.

The pH dependent ζ-potential and particles size profiles provided further information regarding grafting of PLA onto the HA. The weighted average value (*n* = 3) of median particle size and ζ-potential of the non-treated HA and PLA grafted HA (HA-PLA) are shown in Figs. 3A and 3B, respectively. At pH 13, HA presented a negative ζ-potential (Fig. 3A). These negative net surface charges prevented the agglomeration of HA, resulting in an average particle size of approximately 4 μm. Titration from pH 13 to pH 4 led to a gradual change of potential from −25.1 mV to −0.6 mV (Fig. 3A). Due to the decreasing ζ-potential, HA particles tend to conglomerate, resulting in an increase in the average size of HA from 4 μm (pH 13) to 12 μm (pH 4). The sudden decrease in the size of HA below pH 4 can be attributed to the significant dissolution of HA in the highly acidic solution.

Unlike HA, the HA-PLA showed distinctly different pH dependent ζ-potential and particle size profiles (Fig. 3B). At pH 13, HA-PLA and HA present similar negative ζ-potential. However, upon titration of acid, ζ-potential of HA-PLA shows a positive peak (−5.43 mV) at around pH 8, corresponding to the least net surface charge. Further acid titration led to increases in negative charges and reached a peak at pH 5, which then decreased with further decreases in pH. The mechanism of such a change in ζ-potential is not clear and needs to be further studied. Interestingly, the mean size of HA-PLA remained nearly constant between pH 11 and pH 5, suggesting that the PLA coating significantly altered the surface charge and agglomeration properties of HA. It appears that the association of –COO^−^ with H^+^ at below pH 5 reduced the net surface charge of HA-PLA, causing particle agglomeration and increasing the particle size from 4 μm at pH 5 to 8 μm at pH 2. Apparently, the PLA coating protected the HA from rapid dissolution in strong acidic environments.

### 3.4 Improvements in Diametral Tensile Strength (DTS) of PLA-HA Composites Produced by PLA-Grafted HA

**Fig. 4:**
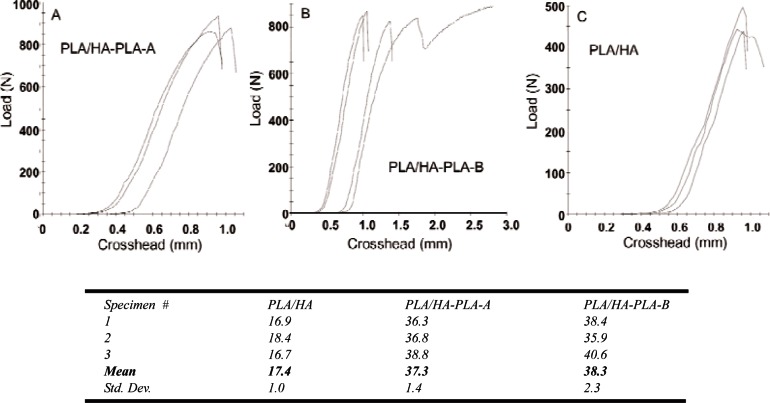
DTS load-strain curves and the corresponding mean DTS values (MPa) of the composites prepared from non-treated HA (PLA/HA), PLA grafted HA by method A (PLA/HA-PLA-A) and method B (PLA/HA-PLA-B), respectively.

Figure 4 shows the load-strain curves and corresponding DTS values for the PLA/HA composites. PLA/HA composite from non-treated HA was (17.4 ± 1.0) MPa (mean ± standard deviation; *n* = 3), which is significantly lower than that (30.3 MPa, unpublished data) of the PLA alone samples prepared from the same polymer. The decrease in strength, which is in agreement with the literature results, can be attributed to the weak interfacial adhesion between the PLA matrix and the non-treated HA.

In contrast, the DTS of the two composites prepared from interfacially improved HA (PLA/HA-PLA-A and PLA/HA-PLA-B) were (37.3 ± 1.4) and (38.3 ± 2.3) MPa, respectively. These values are more than twice (p < 0.05) that of the composite from non-treated HA (17.4 MPa) and 23 % higher than that of the PLA itself. The increased DTS values are also higher than the DTS values of composites with a similar HA ratio reported in the literature [15]. These results imply that combination of SIP with the phosphonic acid agent can significantly improve the tensile strength of the PLA/HA composite. Because HIMPA can lead to a strong interfacial binding between HA and non-ionic –OH groups of HIMPA, the PLA initially grated on the coated-HA can be considered as covalently bond to HA, and the mechanical properties of PLA/HA can be significantly improved.

Nevertheless, statistical analysis did not show a significant difference between the DTS values of the two composites from method A (PLA/HA-PLA-A) and method B (PLA/HA-PLA-B). Further studies are needed to understand more clearly the relationship between the amount of HIMPA coating, the amount of initially grated PLA by SIP, and the mechanical strengths of the composites.

## 4. Discussion

**Fig. 5:**
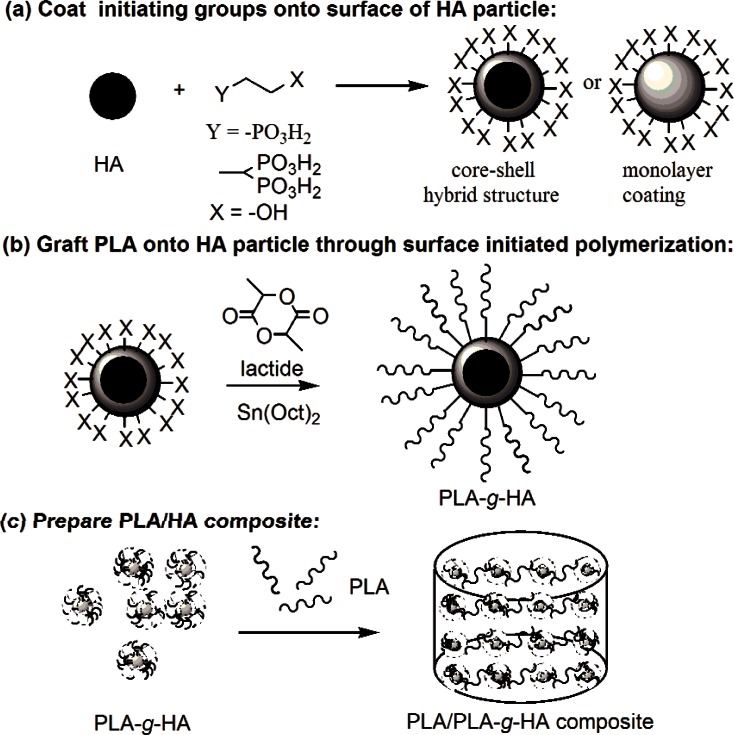
A schematic representation of PLA/HA composite preparation using surface initiated polymerization.

The present study was conducted to assess the feasibility of improving the interfacial interactions between PLA and HA by grafting PLA directly from the surface of HA via surface initiated polymerization (SIP). This approach consists of several conceptual steps as depicted schematically in Fig. 5. The lactide monomer was initiated by the non-ionic –OH groups on the surface of HA in the presence of Sn(Oct)_2_ as the catalyst (Fig. 5b). Because literature data indicated that the innate hydroxyl groups (–OH) on the surface of HA may not be sufficiently reactive and the amount of PLA that can be grafted was limited [15, 32], modification of the HA surface to increase the amount and the type of –OH groups with greater reactivity was necessary to improve PLA grafting. Previous attempts to increase –OH groups on HA through silane and carboxyl functional groups have met limited success [21, 37]. Results from biomineralization and dental materials related studies suggest that phosphonate groups are highly compatible with HA and should have stronger affinity to HA than does silane or carboxyl [22–25]. Thus, in the present study, HIMPA, a molecule with two phosphonic acid groups on one end and an –OH group on the other end, was used as a bidentate chelating agent to link the non-ionic hydroxyl groups to HA (Fig. 5a). It was anticipated that HIMPA may lead to a stronger binding with HA, and a higher amount of HIMPA can be coated on HA than that of mono-phosphonic acid molecule.

Of the two approaches used in the present study to attach non-ionic –OH groups on HA particles, method B, an *in situ* co-precipitation method, in which HA was precipitated in the presence of HIMPA, led to a significantly higher HIMPA incorporation than that by method A. The resulted HIMPA coated HA from method B showed a lower crystallinity than that from method A and standard HA (Fig. 1), suggesting that some phosphonate ions were embedded within the HA crystal lattice, possibly forming a hybrid shell of Ca/(PO_4_^3−^ + R–PO_3_^2−^).

Initiated by the surface non-ionic hydroxyl groups from the HIMPA coating, PLA could be successfully grafted from HA by using surface initiated polymerization in the presence of SnOct_2_. Because ring-opening polymerization of lactide (LA) is sensitive to moisture or impurities in the solvent or reactants, all the solvents including toluene and DMF must be dried over sodium or calcium hydride, respectively. Due to the reactivity of the surface –OH from HIMPA coated HA, the amount of grafted PLA either from method A or method B was greater than that of non-modified HA. In particular, the greater amount of HIMPA coating formed on HA by method B led to a higher amount of grafted PLA (7 % mass fraction), which was higher than that reported in the literature (5 %) [15].

The combination of phosphonic acid coupling agent and surface initiated polymerization facilitated the PLA to link with the HIMPA coated HA surface through covalent bonding, and a strong interfacial adhesion can thus be established. Due to the improved interface and the entanglement of the PLA on the surface of HA and the PLA matrix, the mechanical properties of PLA/HA composites prepared from PLA-grafted HA was significantly improved in comparison to that of the composite prepared using non-grafted HA (17 MPa). This clearly indicates that the interfacial improvement plays a critical role in the mechanical properties of the composite. Despite the significantly higher HIMPA coating produced by method B than that by method A, the mechanical strengths of the two composites were not significantly (*p* > 0.05) different (Fig. 4). This suggests that additional factors, such as the mechanical properties of the HIMAP coated HA particles, may play a role in strengths of the composite.

The design of biocomposites is more complex than that of conventional monolithic materials because of the large number of design variables that must be considered. The mechanical properties of PLA/HA composite are affected by the inherent characteristic of PLA such as chemical configuration, crystallinity, relative molecular mass and polydispersity index, and characteristics of the HA filler such as morphology (particulate or whisker), size distribution, crystallinity (amorphous or crystalline), preparation method (sintered or solution precipitated). Additional important factors include mass fraction of HA, composite preparation methods (solvent casting, hot pressing, compression molding, melt extrusion, biomimetic process, etc.), interfacial treatment as well as specimens fabrication techniques and conditions (heat pressing, casting, sintering, machining, together with molding temperature, pressure, and processing time). In order to specifically understand the effect of interfacial improvement proposed in this research, the composites for this study were prepared from the same PLA under the same experimental condition, e.g., filler ratio, composite technology, temperature, molding pressure, etc., with the interfacial optimization of the HA particles (non-treated HA or PLA grafted HA) being the only difference. Due to its limited scope, the present study did not produce a PLA-HA composite with strengths sufficiently high for use in load-bearing bone repair applications. However, the results demonstrated the importance of improving the interfacial adhesion in developing stronger PLA-HA composite materials.

## 5. Summary

A bi-phosphonic acid chelating agent was used to attach firmly bound hydroxyl groups to the surfaces of HA particles. An *in situ* co-precipitation method (method B) that formed HA with a calcium-phosphate/phosphonate hybrid shell configuration seemed to be a more efficient approach to coat a greater amount of the chelating agent on HA particles than that produced by coating a monolayer of the phosphonic acid (method A). PLA can be successfully grafted on HA through surface initiated polymerization initiated by the non-ionic surface hydroxyl groups on the chelated HA particles. The greater amount of phosphonic acid on HA led to a higher amount of grafted PLA that is covalently linked to the HA surface, resulting in a stronger interfacial adhesion between the PLA and HA. The PLA coating was found to significantly alter the surface characteristics of the HA particles in terms of both the surface charge and particle agglomeration. The tensile strengths of PLA/HA composites prepared from PLA-grafted HA were found to be over twice that of the composite prepared from conventional non-modified HA, and were 23 % higher than that of PLA alone. These results indicate that a combination of SIP with this phosphonic acid chelating agent can significantly improve the tensile strength of the PLA/HA composite. Optimization of additional variables, including molecular weight of PLA, size and composition of calcium phosphate filler, end-group cross-linking, etc., should lead to further improvements in mechanical strengths of the PLA-HA composites in the range of natural bone.

## Footnotes

1 Certain commercial materials and equipment are identified in this paper to specify the experimental procedure. In no instance does such identification imply recommendation or endorsement by the National Institutes of Health, American Dental Association Foundation, or National Institute of Standards and Technology or that the material or equipment identified is necessarily the best available for the purpose.
